# NT-proBNP and Its Correlation with In-Hospital Mortality in the Very Elderly without an Admission Diagnosis of Heart Failure

**DOI:** 10.1371/journal.pone.0153759

**Published:** 2016-04-14

**Authors:** Riccardo Sarzani, Francesco Spannella, Federico Giulietti, Massimiliano Fedecostante, Piero Giordano, Pisana Gattafoni, Emma Espinosa, Franco Busco, Gina Piccinini, Paolo Dessì-Fulgheri

**Affiliations:** 1 Italian National Research Centre on Aging “U.Sestilli”, IRCCS-INRCA, Ancona, Italy; 2 Internal Medicine and Geriatrics, Department of Clinical and Molecular Sciences, University “Politecnica delle Marche”, Ancona, Italy; 3 Clinical Analysis Laboratory, Italian National Research Centre on Aging “U.Sestilli”, IRCCS-INRCA, Ancona, Italy; University of Naples Federico II, ITALY

## Abstract

**Background:**

The diagnosis of heart failure (HF) is often difficult and underestimated in very elderly comorbid patients, especially when an echocardiographic evaluation is not available or feasible. Aim: to evaluate NT-proBNP values and their correlation with in-hospital mortality in a population of very elderly hospitalized for medical conditions other than HF.

**Methods:**

We performed a prospective observational study on 403 very elderly admitted to an Internal Medicine and Geriatrics Department. Exclusion criterion was an admission diagnosis of HF. Patients with at least one symptom or sign compatible with HF were tested for NT-proBNP. NT-proBNP values < 300 pg/ml were considered as an age-independent exclusion criterion for HF (high negative predictive value), while NT-proBNP values ≥ 1800 pg/ml were considered as a diagnostic criterion. Main comorbidities and laboratory parameters were considered to adjust regression analyses between NT-proBNP and in-hospital mortality.

**Results:**

NT-proBNP values ≥ 1800 pg/ml were present in 61.0% of patients and 32.8% of patients laid between 300 ≤ NT-proBNP < 1800 pg/ml values. NT-proBNP values were associated with the main indices of disease severity/organ failure considered such as reduced eGFR, reduced albumin and elevated CRP. NT-proBNP values ≥ 1800 pg/ml and ln(NT-proBNP) values were significantly associated with in-hospital mortality independently from the main comorbidities and lab parameters considered. The patients, who were already taking ACE inhibitors/Angiotensin Receptor Blockers before admission, showed lower in-hospital mortality.

**Conclusions:**

Testing for NT-proBNP should be strongly recommended in the hospitalized very elderly, because of the very high prevalence of underlying HF and its impact on in-hospital mortality, to identify an underlying cardiac involvement that requires appropriate treatment.

## Introduction

Heart failure (HF) is a major health concern. It is highly prevalent and it is associated with high morbidity, high mortality and considerable healthcare costs. HF is the first cause of hospitalization in older patients because aging leads to the increasing prevalence of HF with epidemic-like aspects in octogenarian people [1,2]. In Italy, HF causes about 200,000 hospitalizations per year (88% among people aged > 65 years) with an increasing trend [3]. Aging is associated with a set of cardiac modifications such as reduction of the number of cardiomyocytes, hypertrophy of residual cardiomyocytes, perivascular and interstitial fibrosis, reduced release of calcium from calcium-dependent contractile proteins and delayed reuptake of intracellular calcium from the sarcoplasmic reticulum. These changes lead to decreased ventricular compliance predisposing to the development of HF, mainly with preserved ejection fraction [4], frequently secondary to hypertension with left ventricular hypertrophy.

Despite the very elderly population is steadily increasing, it is almost always excluded from RCT and studies on HF, leading to an important gap in published evidence including diagnostic guidelines. HF diagnosis is traditionally based on history and complete physical examination. However, in the very elderly, it can be very difficult to obtain a clear history and the clinical picture is often confounded by comorbidities. Moreover, the combination of inactivity and comorbidity in the very elderly may mask the typical clinical symptoms and signs of HF, such as dyspnea, easy fatigability and leg edema. Exertional dyspnea for example, one of the cardinal symptoms of HF, may not be reported because of reduced physical activity or because it may be attributed to other comorbidities such as chronic lung disease, anemia and kidney failure [5].

Moreover, it is important to emphasize that, in the context of a reduced functional heart reserve in very elderly patients, the insurgence of an acute disease can unveil the latent HF, leading to a full-blown clinical picture.

In an internal medicine/geriatrics setting, the availability of echocardiographic evaluation is often limited [6]. Thus, it is essential to adopt a non-operator-dependent test with a good sensitivity/specificity profile and an affordable cost, to confirm or rule out a diagnosis of HF and also to assess its severity and prognosis. The dosage of NT-proBNP is a well-established affordable test for the exclusion or confirmation of HF, guidance of therapy and prognosis. European HF Guidelines indeed recommend the dosage of natriuretic peptides (NP) in patients presenting with acute onset or worsening of symptoms or signs compatible with HF and the optimal exclusion cut-off point is 300 pg/ml for NT-proBNP and 100 pg/ml for BNP.[1] Although elevated NT-proBNP levels may reflect a functional and structural cardiac impairment, the interpretation of the results can be confounded by the presence of other conditions, such as renal dysfunction, atrial fibrillation and anemia that are typical of the older people. However, elevation of NT-proBNP levels in the context of non-HF situations should not be considered just as a “false-positive” result, because of the serious adverse outcomes associated with elevated NT-proBNP levels. [7, 8] The observation of a direct relationship between age and levels of NT-proBNP, likely consequent to age-related changes in left ventricular compliance as well as decreasing eGFR and presence of atrial fibrillation, support the importance of age-adjusted NT-proBNP cut-offs.

The most validated and widely used age-adjusted cut-off for HF diagnosis is a value of 1800 pg/ml in patients aged 75 years and older. [9]

However, few studies have assessed the role of NT-proBNP dosage in the very elderly. In the context of periodical check of our clinical protocols for “best clinical practice”, a retrospective survey showed that NT-proBNP levels were frequently elevated in the very elderly, even when HF was not suspected at first and was with fewer symptoms and without specific symptoms/signs.

Therefore, the aim of this prospective observational study was to evaluate NT-proBNP values and their association with comorbidities, disease severity and in-hospital mortality, in a population of very elderly hospitalized for medical conditions other than HF.

## Materials and Methods

We analyzed data of 403 very elderly consecutively admitted to an Internal Medicine and Geriatrics Department, from January 2013 to December 2014, where NT-proBNP was evaluated in the attempt to ensure a “best clinical practice” approach. An admission diagnosis of HF confirmed by a cardiologist, was considered as an exclusion criterion. All other patients with at least one symptom or sign compatible with HF were tested for NT-proBNP. We also considered the following laboratory parameters: hemoglobin, white blood cells count, creatinine, estimated Glomerular Filtration Rate (eGFR), serum sodium and potassium, glycaemia, C reactive protein, albumin, total cholesterol.

Creatinine was determined in serum or plasma using a “Creatinine Jaffé of second generation in a Cobas c 501 Roche analyzer” and the eGFR was estimated using the CKD-EPI equation. [10]

To evaluate patients’ functional status, the seven point MDS Activities of Daily Living (ADL) Hierarchy scale was used. [11] The ADL Hierarchy scale groups activities of daily living according to the stage of the disablement process in which they occur. The ADL Hierarchy Scale ranges from 0 (no dependence) to 6 (total dependence). ADL disability was categorized as follows: no impairment (ADL Hierarchy Scale score <2), assistance required (ADL Hierarchy Scale score 2 to 4) and dependence (ADL Hierarchy Scale score ≥ 5). Baseline CRP dosage was present (because of clinical significance in the patients management) in 318 patients. In the statistical analysis, chronic bedridden (defined as a bedridden status for more than 3 months) was considered as condition at greater mortality risk and the history of HF as a possible cause of elevated baseline levels of NT-proBNP. Polypharmacy was defined as the use of five or more drugs. The Mini-Cog, which combines two simple cognitive tasks (three-item word memory and clock drawing) with an empirical algorithm for scoring, was performed to evaluate cognitive impairment. [12] The Geriatric index of comorbidity (GIC) was used to determine the burden of comorbidities [13] and it was categorized as low comorbidity (GIC classes 1–2) and high comorbidity (GIC classes 3–4).

### NT-proBNP assay

After blood sampling, NT-proBNP was measured using "Elecsys proBNPII electrochemiluminescence Immunoassay in a Cobas e 601 immunoassay Roche analyzer”. This assay contains two monoclonal antibodies which recognize epitopes located in the N-terminal part (1–76) of proBNP (1–108). We considered the age-adjusted cut-offs proposed by Januzzi et al, because they are the most widely used and validated thresholds in clinical practice and also recommended by our laboratory of clinical chemistry. NT-proBNP < 300 pg/ml was considered as an age-independent exclusion criterion for HF (high negative predictive value), while NT-proBNP values ≥ 1800 pg/ml were considered diagnostic for HF. NT-proBNP values between 300 pg/ml and 1799 pg/ml were considered as a “gray zone”. The cut-off of 1800 pg/ml does not require further adjustment for renal function and remains useful for the diagnostic and prognostic evaluation of patients with CKD. [7]

Clinical investigations have been conducted according to the principles expressed in the Declaration of Helsinki. Data were collected and analyzed anonymously, so it was not necessary to obtain informed consent from each patient. This observational study of a “best clinical practice” experience was approved by the local institutional ethics committee (“Comitato Etico Regionale delle Marche”—CERM).

### Statistical analysis

Data were analyzed with the Statistical Package for Social Science version 13 (SPSS Inc. Chicago, Illinois, USA). A value of p < .05 was defined as statistically significant. All continuous variables were expressed as mean ± SD, except NT-proBNP that was expressed as median and interquartile range, because it was markedly skewed. Moreover, NT-proBNP was considered as a continuous variable and was natural logarithmically transformed (ln NT-proBNP) to normalize its distribution. Categorical variables were expressed as absolute number and percentage. Wilcoxon signed rank test was used to compare NT-proBNP values at admission and at discharge. Cross-sectional analyses were undertaken to examine associations of NT-proBNP values with comorbidities and laboratory parameters as well as to examine associations between all of these factors and mortality. Associations identified on univariate analyses with p < .10 were tested in multivariate regression models; covariables were included in the analysis on identification of an association on univariate analyses.

## Results

General characteristics of the studied population are shown in Table 1, stratified by NT-proBNP cut-offs.

**Table 1:** General Characteristics.

Clinical Characteristics	All patients (n° 403)	NT-proBNP < 300 pg/ml (n° 25)	300 ≤ NT-proBNP < 1800 pg/ml (n° 132)	NT-proBNP ≥ 1800 pg/ml (n° 246)	p
**Age (years)**	**88.1 ± 5.1**	**86.7 ± 4.6**	**87.1 ± 5.0**	**88.8 ± 5.1**	**.003**
Sex: female	230 (57.1%)	18 (72%)	71 (53.8%)	141 (57.3%)	.239
Length of stay (days)	11.2 ± 6.7	11.6 ± 6.3	11.9 ± 6.9	10.8 ± 6.6	.289
**Polypharmacy**	**295 (73.2%)**	**17 (68%)**	**81 (61.4%)**	**197 (80.1%)**	**<.001**
Cognitive impairment	188 (46.7%)	10 (40%)	61 (46.2%)	117 (47.6%)	.765
GIC: Low comorbidity	45 (11.2%)	4 (16%)	21 (15.9%)	20 (8.1%)	.053
GIC: High comorbidity	358 (88.8%)	21 (84%)	111 (84.1%)	226 (91.9%)	.053
ADL Hierarchy Scale: No impairment	97 (24.1%)	6 (24%)	36 (27.3%)	55 (22.4%)	.647
ADL Hierarchy Scale: Assistance required	146 (36.2%)	11 (44%)	48 (36.4%)	87 (35.4%)	.647
ADL Hierarchy Scale: Dependence	160 (39.7%)	8 (32%)	48 (36.4%)	104 (36.4%)	.647
**Systolic BP (mmHg)**	**123.6 ± 25.3**	**127.1 ± 25.5**	**127.9 ± 25.2**	**121.0 ± 25.1**	**.031**
Diastolic BP (mmHg)	70.3 ± 13.3	73.0 ± 13.2	71.4 ± 12.9	69.3 ± 13.5	.195
Heart Rate (bpm)	82.2 ± 16.8	75.2 ± 15.5	81.7 ± 17.2	83.2 ± 16.6	.072
**Admission diagnoses** *					
COPD exacerbation	121 (30%)	7 (28%)	36 (27.3%)	78 (31.7%)	.652
Pneumonia	98 (24.3%)	6 (24%)	29 (22%)	63 (25.6%)	.733
UTI or other infections	91 (22.6%)	6 (24%)	27 (20.5%)	58 (23.6%)	.775
Acute kidney injury	77 (19.1%)	4 (16%)	17 (12.9%)	56 (22.8%)	.061
Advanced cancer	47 (11.7%)	3 (12%)	12 (9.1%)	32 (13%)	.527
**Atrial fibrillation**	**109 (27.0%)**	**4 (16%)**	**20 (15.2%)**	**85 (34.6%)**	**<.001**
**Admission lab parameters**					
**NT-proBNP (pg/ml)**	**2404 (25°-75° pcs: 996–6446)**	**172 (25°-75° pcs: 112–245)**	**891 (25°-75° pcs: 584–1379)**	**5330 (25°-75° pcs: 2821–11422)**	**<.001**
**Hgb (g/dl)**	**11.5 ± 2.1**	**12.4 ± 2.0**	**11.8 ± 2.2**	**11.2 ± 2.1**	**.004**
WBC (n/mm^3^)	11332 ± 6077	9558 ± 3860	11344 ± 5966	11507 ± 6306	.312
**eGFR (ml/min/1.73 m**^**2**^**)**	**48.3 ± 24.1**	**56.6 ± 24.4**	**56.7 ± 23.2**	**42.9 ± 23.1**	**<.001**
Serum sodium (mEq/l)	136.9 ± 7.1	136.3 ± 5.7	137.4 ± 7.4	136.8 ± 7.1	.682
Serum potassium (mEq/l)	4.2 ± 0.8	4.2 ± 0.6	4.1 ± 0.7	4.2 ± 0.8	.735
Glycaemia (mg/dl)	135.0 ± 62.3	125.3 ± 49.1	138.0 ± 72.3	134.4 ± 57.6	.633
**Albumine (g/dl)**	**3.2 ± 0.6**	**3.3 ± 0.5**	**3.3 ± 0.6**	**3.1 ± 0.6**	**.001**
**Total cholesterol (mg/dl)**	**142.8 ± 39.5**	**155.1 ± 46.4**	**150.9 ± 42.8**	**137.1 ± 35.9**	**.002**
**CRP (mg/dl)** ¥	**8.89 ± 8.51**	**4.66 ± 3.99**	**7.04 ± 6.89**	**10.12 ± 9.22**	**.002**
**Cardiovascular therapy before admission**					
ACE-I/ARBs	166 (41.2%)	13 (52%)	48 (36.4%)	105 (42.7%)	.259
**Diuretics**	**249 (61.8%)**	**16 (64%)**	**61 (46.2%)**	**172 (69.9%)**	**<.001**
**Beta blockers**	**126 (31.1%)**	**3 (12%)**	**26 (19.7%)**	**97 (39.4%)**	**<.001**
Calcium channel blockers	60 (14.9%)	4 (16%)	22 (16.7%)	34 (13.8%)	.750
Mineralocorticoid antagonists	88 (21.8%)	6 (24%)	21 (15.9%)	61 (24.8%)	.132
Antiplatelet drugs	162 (40.2%)	10 (40%)	51 (38.6%)	101 (41.1%)	.900
Statins	108 (26.8%)	7 (28%)	38 (28.8%)	63 (25.6%)	.794
Anticoagulants	127 (31.5%)	7 (28%)	32 (24.2%)	88 (35.8%)	.066

All continuous variables were expressed as mean ± SD, except NT-proBNP that was expressed as median and interquartile range, because it was markedly skewed. Categorical variables were expressed as absolute number and percentage. Polypharmacy was defined as the use of five or more drugs.UTI: urinary tract infection; Hgb: hemoglobin; WBC: white blood cells; eGFR: estimated glomerular filtration rate; CRP: C reactive protein; BP: blood pressure; ACE-I: angiotensin-converting enzyme (ACE) inhibitors; ARBs: angiotensin II receptor blockers.

*Each patient could have more than one admission diagnosis.

^¥^ CRP was evaluated in 318 patients.

Mean age was 88.1 ± 5.1 years (range: 80–103 years), with female prevalence (57.1%). Patients with NT-proBNP values ≥ 1800 pg/ml at admission had older age, lower systolic blood pressure, lower hemoglobin values, lower eGFR, lower levels of albumin and total cholesterol and higher levels of C-reactive protein compared to patients with NT-proBNP values < 1800 pg/ml (all p < .05). Patients with NT-proBNP values ≥ 1800 pg/ml were taking a higher number of drugs (p < .001) and they also seemed to have a higher burden of comorbidities, however without reaching the statistical significance.

No statistical differences were found in the cognitive and functional status. The leading causes of hospitalization and the main cardiovascular therapies taken by patients before admission are also summarized in Table 1. No differences in admission diagnoses between NT-proBNP groups were found, except for AF, which was more prevalent in patient with NT-proBNP ≥ 1800 pg/ml (p < .001).

### Prevalence of HF and its association with admission diagnoses and clinical lab parameters

Only 78 patients (19.4%) had a history of HF before hospitalization, although HF was not the admission diagnosis.

NT-proBNP values ≥ 1800 pg/ml were present in 246 patients (61.0%) whereas patients in the “gray zone” (300 ≤ NT-proBNP < 1800 pg/ml) were 32.8%. Therefore, NT-proBNP values ≥ 1800 pg/ml were greatly prevalent regardless of the admission diagnosis.

Comorbidities and laboratory parameters that showed an association with NT-proBNP values at univariate analysis with a p < .10 were included in a logistic regression model (for the dichotomic variable HF) and in a multiple linear regression model (for ln-transformed NT-proBNP) to test their independent association and their relevance. Data are shown in Tables 2 and 3. Estimated-GFR and atrial fibrillation showed the better relation with NT-proBNP, followed by polypharmacy, CRP, albumin and age.

**Table 2:** Logistic Regression for HF (NT-proBNP ≥ 1800 pg/ml).

Variables*	Model 1			Model 2		
	Wald	OR (95% CI)	p	Wald	OR (95% CI)	p
History of HF	**12.6**	**3.84 (1.83–8.06)**	**<.001**	**8.9**	**4.03 (1.65–11.27)**	**.003**
AF	**10.6**	**2.65 (1.47–4.77)**	**.001**	**12.7**	**3.71 (1.81–7.63)**	**<.001**
Age	**5.6**	**1.06 (1.01–1.12)**	**.018**	**5.2**	**1.07 (1.01–1.14)**	**.023**
CRP				**7.4**	**1.06 (1.02–1.10)**	**.007**
eGFR	**14.3**	**0.98 (0.97–0.99)**	**<.001**	**11.2**	**0.98 (0.96–0.99)**	**.001**
Albumin	**7.2**	**0.50 (0.31–0.83)**	**.007**	**4.5**	**0.51 (0.27–0.95)**	**.034**
AKI	0.0	1.03 (0.51–2.09)	.935	1.2	1.62 (0.69–3.79)	.268
Total cholesterol	1.6	1.00 (0.99–1.00)	.208	0.0	1.00 (0.99–1.01)	.993
Hgb	1.4	0.93 (0.82–1.05)	.240	1.8	0.90 (0.77–1.05)	.179
GIC (ref: Low)	0.2	0.84 (0.40–1.77)	.645	1.1	0.63 (0.26–1.50)	.296
Heart rate	0.5	1.01 (0.99–1.02)	.499	0.1	1.00 (0.99–1.02)	.715
Polypharmacy	**7.98**	**2.24 (1.28–3.93)**	**.005**	**7.6**	**2.62 (1.32–5.20)**	**.006**
Systolic BP	0.7	1.00 (0.99–1.05)	.397	1.3	0.99 (0.98–1.01)	.254

* For continuous variables OR was for a one unit increase.

Model 1 included all the 403 patients.

Model 2 included the 318 patients with the basal CRP evaluation.

AKI: acute kidney injury (as an admission diagnosis); AF: atrial fibrillation; Hgb: hemoglobin; eGFR: estimated glomerular filtration rate; CRP: C reactive protein.

**Table 3:** Multiple Linear Regression for ln(NT-proBNP).

Variables**	Model 1			Model 2		
	beta	B (95% CI)	P	beta	B (95% CI)	p
History of HF	**0.197**	**0.69 (0.37–1.01)**	**<.001**	**0.226**	**0.81 (0.45–1.16)**	**<.001**
AF	**0.207**	**0.64 (0.36–0.92)**	**<.001**	**0.262**	**0.78 (0.49–1.07)**	**<.001**
Albumin	**-0.124**	**-0.29 (-0.53–-0.05)**	**.018**	**-0.147**	**-0.33 (-0.59–-0.07)**	**.014**
Age	**0.129**	**0.04 (0.01–0.06)**	**.005**	**0.134**	**0.03 (0.01–0.06)**	**.007**
Heart rate	**0.102**	**0.01 (0.00–0.02)**	**.024**	0.088	0.01 (0.00–0.01)	.072
CRP				**0.134**	**0.02 (0.01–0.04)**	**.009**
eGFR	**-0.294**	**-0.02 (-0.02–-0.01)**	**<.001**	**-0.264**	**-0.02 (-0.02–-0.01)**	**<.001**
AKI	-0.061	-0.22 (-0.57–0.14)	.228	-0.003	-0.01 (-0.37–0.35)	.951
Hgb	**-0.099**	-**0.07 (**-**0.13–0.00)**	**.041**	-0.089	-0.06 (-0.12–0.01)	.093
Total cholesterol	-0.066	0.00 (-0.01–0.00)	.177	-0.011	0.00 (-0.01–0.00)	.839
GIC (ref: Low)	0.012	0.05 (-0.34–0.45)	.799	0.006	0.02 (-0.38–0.43)	.911
Polypharmacy	**0.104**	**0.33 (0.04–0.61)**	**.024**	**0.107**	**0.33 (0.03–0.63)**	**.033**
Systolic BP	-0.044	0.00 (-0.01–0.00)	.345	**-0.109**	**-0.01 (-0.01–0.00)**	**.029**

** For continuous variables B represented the amount of change in ln-transformed NT-proBNP for a one unit increase in independent variable.

Model 1 included all the 403 patients.

Model 2 included the 318 patients with the basal CRP evaluation.

AKI: acute kidney injury (as an admission diagnosis); AF: atrial fibrillation; Hgb: hemoglobin; eGFR: estimated glomerular filtration rate; CRP: C reactive protein.

### In-hospital mortality

**Fig 1:**
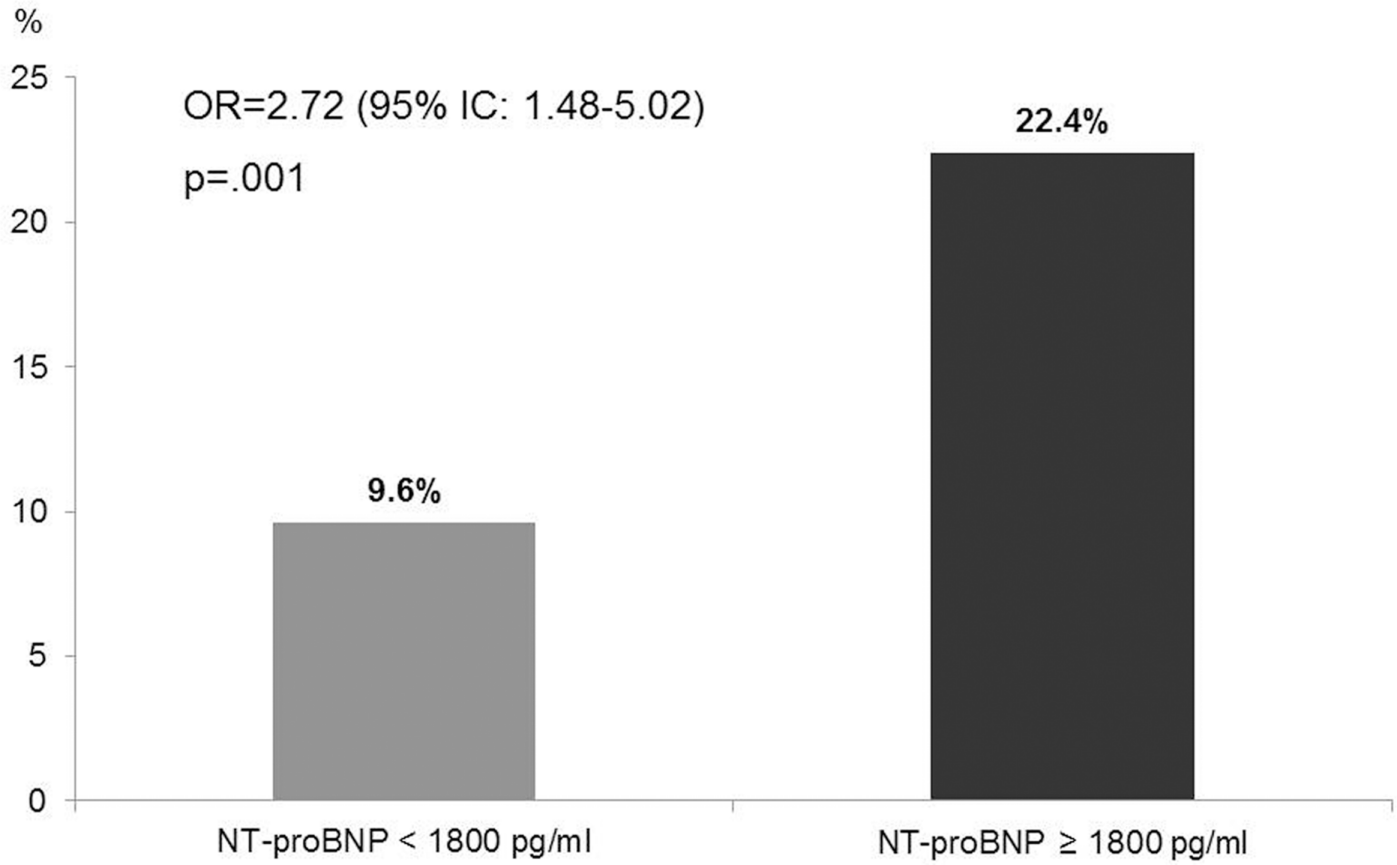
In-hospital mortality according to NT-proBNP levels.

Seventy patients (17.4%) died during hospitalization. A cardiac cause of death was documented in 56.4% of patients with an admission NT-proBNP value ≥ 1800 pg/ml. HF, as diagnosed by NT-proBNP levels, showed a positive association with in-hospital mortality: patients with NT-proBNP ≥ 1800 pg/ml had an OR = 2.72 (95% CI: 1.48–5.02; p = .001) for in-hospital mortality (Fig 1). The variables associated with mortality on univariate analysis were included in a logistic regression model with NT-proBNP: high values of NT-proBNP confirmed their association with poor outcome (death) (Table 4). Among the multiple cardiovascular therapies taken before admission, only ACE-I/ARBs showed an association with mortality (OR = 0.47 (95% CI: 0.23–0.95; p = .037) after adjusting for advanced cancer, bedridden status, WBC, age, glycaemia, HF, heart rate, albumin.

**Table 4:** Logistic Regression for In-hospital Mortality.

Variables	Model 1			Model 2		
	Wald	OR (95% CI)	p	Wald	OR (95% CI)	p
Advanced cancer	**14.8**	**4.60 (2.12–10.02)**	**<.001**	**15.0**	**4.70 (2.15–10.30)**	**<.001**
Heart failure	**3.9**	**2.04 (1.01–4.14)**	**.049**			
ln(NT-proBNP)				**6.2**	**1.35 (1.07–1.70)**	**.013**
Age	**10.5**	**1.10 (1.04–1.17)**	**.001**	**9.6**	**1.10 (1.04–1.17)**	**.002**
Glycaemia	**4.1**	**1.01 (1.00–1.01)**	**.043**	**4.7**	**1.01 (1.00–1.01)**	**.031**
Albumin	**19.4**	**0.29 (0.17–0.50)**	**<.001**	**19.6**	**0.29 (0.17–0.50)**	**<.001**
WBC	0.4	1.0 (1.0–1.1)	.552	0.2	1.0 (0.96–1.06)	.678
Chronic bedridden	2.7	1.7 (0.9–3.3)	.104	2.5	1.70 (0.88–3.28)	.114
Heart rate	1.3	1.0 (0.9–1.0)	.264	0.9	1.01 (0.99–1.03)	.355

For continuous variables OR was for a one unit increase. WBC: white blood cell. Heart failure was defined as an NT-proBNP ≥ 1800 pg/ml.

## Discussion

Our study shows that, in the very elderly hospitalized with an admission diagnosis different from HF, this condition, as evidenced by NT-proBNP values ≥ 1800 pg/ml, had a very high prevalence and frequently complicated the clinical scenario and affected in-hospital mortality, regardless the main diagnosis of presentation.

The very elderly may present atypical and non- specific symptoms/signs of HF such as cough, tiredness, ankle swelling, weight loss, general malaise, that may also be due to one or more comorbidities often present, such as respiratory and renal diseases.

Even dyspnea is not specific and it may be difficult to stratify in the very elderly using a static approach. [14, 15] Moreover, the very elderly with chronic HF may have different causes of decompensation than younger and most of the time multiple factors coexist, such as infections and hyperkinetic arrhythmias, thus confirming that comorbidities may influence the cardiac function in this population. [16] In this clinical scenario, NT-proBNP dosing may be helpful to evaluate the cardiac involvement.

In our study, 61% of patients had NT-proBNP values compatible with a diagnosis of HF (considering the age-related cut-off of NT-proBNP ≥ 1800 pg/ml), while a significant portion of patients (32.8%) laid in the "gray zone", with NT-proBNP values between 300 and 1799 pg/ml, that did not exclude the presence of HF and could highlight significant underlying cardiac structural and functional abnormalities. [7]

Our findings are very relevant especially considering that only 78 patients (19.4%) had a previous history of HF.

These results are better interpretable when considering that most of very elderly individuals probably have a broad array of significant underlying cardiac functional and structural abnormalities often due to decades of superimposed cardiovascular risk factors [17]: when an acute intercurrent illness increases body’s metabolic demands, these abnormalities facilitate the development of HF. Indeed, NT-proBNP values appeared to be associated with the main indices of disease severity/organ failure considered such as reduced eGFR, reduced albumin, elevated CRP and polypharmacy, that can be considered an indirect measure of the patient's burden of comorbidities.

This was not only a prevalence finding: our data clearly showed that higher NT-proBNP values correlated with in-hospital mortality after adjusting for covariates clinically and significantly associated with mortality (such as advanced cancer, chronic bedridden and age), similarly to the findings on this ventricular natriuretic peptide in younger populations [14, 18]. In other words, most of the very elderly with an acute illness have also a cardiac involvement, as evidenced by NT-proBNP values, and, more important, higher NT-proBNP values are closely associated with mortality, regardless the “triggering” disease.

Regarding the prognostic importance of NT-proBNP values, Di Somma et al. showed similar results for BNP levels: in their studies the variation of BNP levels during hospitalization and BNP levels assessed 30 days after hospital discharge were predictive of subsequent hospitalization and death in an elderly population, also in the absence of signs and symptoms compatible with HF. [19, 20, 21]

European HF Guidelines recommend echocardiography to evaluate cardiac structure and function, including diastolic function and to measure LVEF to make the diagnosis of HF, assist in planning and monitoring of treatment, and to obtain prognostic information. [1]. However, Doppler echocardiography is difficult to interpret in the very elderly, particularly in the presence of atrial fibrillation in which several echocardiographic diastolic components cannot be properly measured and the diagnostic values for diastolic left ventricular dysfunction in patients aged > 80 years are uncertain [22, 23]. In addition, the applicability of current echocardiographic cut-off values to very elderly subpopulations may be debatable since the thresholds for normal values are usually derived from younger subpopulations [24]. Moreover, cardiologic consultation coupled with ready available echocardiographic assessment of heart dimensions and function, is often difficult to obtain promptly, especially considering that most, if not all, of these very elderly patients should ideally undergo echocardiography.

In this context, NT-proBNP is inexpensive and appeared to be a good indicator of cardiac overload/distress, although it is not specific only for left ventricular systolic dysfunction, since it could be elevated in diastolic left ventricular dysfunction or pulmonary disease leading to pulmonary hypertension and right ventricular dysfunction. NT-proBNP is a well known disease-specific marker of cardiac illness and a predictor of mortality, even in a population of very elderly outpatients with a high burden of comorbidity [25]. Increasing levels of NT-proBNP are related to the number of echocardiographic abnormalities and it could possibly be used to identify “pancardiac” damage, even when it is silent [24].

It is known that circulating concentrations of both NT-proBNP and BNP are correlated to the renal function. In the presence of renal disease, the cause of elevated levels of NP seems to be multifactorial and not simply the diminished passive renal clearance. NP levels seem to be only elevated in renal dysfunction when there have been anatomic (e.g., LV hypertrophy) and/or functional (e.g., diastolic dysfunction) changes in the heart. [26] Therefore, high NP levels should not be ignored in the setting of renal dysfunction, given the strong relationship between cardiac and renal disease, and clearly elevated NP values suggest that a cardiac disease is present and should influence clinical decision-making. [8]

At present, the cut-offs to be used for HF diagnosis in the very elderly are still unclearly defined. In a recent study, Bombelli et al. proposed higher NT-proBNP cut-off values compared with the most used cut-offs, showing a gain in specificity and positive predictive value with a slightly lower performance in sensitivity and negative predictive value and with an increase in the “gray zone” of diagnostic uncertainty. [27]

However, in our study the relation between NT-proBNP and in-hospital mortality seems to be continuous, regardless of the cut-offs considered, thus reflecting the importance of NT-proBNP values.

The dosage of NT-proBNP is also useful to guide HF therapy in elderly patients. [28] In our study the recognition of NT-proBNP ≥ 1800 pg/ml at admission conditioned drug therapy during hospitalization, leading to an increase in the prevalence of patients treated with β-blockers and RAS blockers at discharge compared to admission (69% vs 40.3% for β-blockers; 70.1% vs 48.7% for RAS blockers) and a significant reduction of NT-proBNP values at discharge was observed: 5811 pg/ml at admission (25°-75° pcs: 2940.5 pg/ml—14856.5 pg/ml) vs 3498 pg/ml at discharge (25°-75° pcs: 1888 pg/ml—7171 pg/ml); p < .001.

The importance of heart protection in the very elderly to prevent in-hospital mortality also emerged when analyzing data regarding the main cardiovascular therapies before admission: treatment with ACE-I/ARBs, cornerstone drugs in cardiovascular management, showed a protective effect against in-hospital mortality.

In conclusion, in very elderly hospitalized patients, even when a diagnosis different from HF could justify the patient's clinical presentation and symptoms are not specific for HF, a cardiac involvement must be suspected because of the very high prevalence of underlying HF and its impact on in-hospital mortality. Therefore, testing for NT-proBNP should be strongly and widely recommended to identify an underlying cardiac involvement that can be further investigated by echocardiography when available. HF therapy guided by NT-proBNP values may be warranted to ensure better outcomes.

### Study limits

The main limit of our study is the lack of a systematic echocardiographic evaluation for the reasons explained above, a real daily practice condition that stimulated to conduct the present study. Indeed, the focus of our study was on the use of NT-proBNP to detect HF in settings where echocardiography is unavailable, difficult to perform, or potentially unable to provide additional useful clinical information. Nevertheless, in a sub-group of our population (14.3%) a full cardiologic evaluation with echocardiography was performed during hospitalization or at the discharge, with the diagnosis of HF confirmed in all cases. It is important to note that the strong correlation of NT-proBNP level with in-hospital mortality clearly highlights the importance of this test in the very elderly, a key novel finding on very elderly hospitalized with an admission diagnosis different from HF.
